# Association of methylenetetrahytrofolate reductase (*MTHFR*) C677T and A1298C polymorphisms with the susceptibility of childhood acute lymphoblastic leukaemia (ALL) in Chinese population

**DOI:** 10.1186/2047-783X-19-5

**Published:** 2014-01-29

**Authors:** Xiaolei Li, Qingchuan Liao, Shunguo Zhang, Minling Chen

**Affiliations:** 1 Department of Pharmacy, Shanghai Children’s Medical Center, Shanghai Jiaotong University School of Medicine, 1678 Dongfang Road, Pudong, Shanghai 200127, China; 2 Department of Pharmacy, Nanjing Children’s Hospital, Nanjing Medical University, Nanjing 210008, China

**Keywords:** Methylenetetrahydrofolate reductase (MTHFR), C677T, A1298C, Gene polymorphism, Acute lymphoblastic leukemia

## Abstract

**Background:**

The aim of this study was to investigate the relationship between the polymorphisms of the methylenetetrahytrofolate reductase (MTHFR) gene and susceptibility to childhood acute lymphoblastic leukemia (ALL).

**Methods:**

A case–control study was conducted among 98 children with ALL and 93 age- and sex- matched non-ALL controls. Genotyping of *MTHFR* C677T and A1298C polymorphisms was performed by polymerase chain reaction-restriction fragment length polymorphism (PCR-RFLP). The odds ratios (ORs) of *MTHFR* genotypes were used to assess the associations of these polymorphisms with childhood ALL susceptibility.

**Results:**

No significant differences were observed for frequencies of the 677CC, 677CT and 677TT genotypes between patients and controls. Frequencies of the 1298AA, 1298 AC and 1298CC genotypes between the two groups were significantly different. The risk of ALL with the 1298C allele carriers (AC + CC) was elevated by 1.1 times compared with the AA genotype [OR = 2.100; 95% CI (1.149; 3.837); *P* = 0.015].

**Conclusions:**

The *MTHFR* A1298C polymorphism is associated with susceptibility to childhood ALL in the Chinese population.

## Background

Acute lymphoblastic leukemia (ALL) is a malignant disorder of lymphoid progenitor cells with a peak prevalence at the ages 2–5 years old [1] and accounts for one third of all childhood malignancies [2,3]. Although the rapid progress in the clinical diagnosis and effective treatment of childhood ALL has led to a cure rate of more than 80% in children [4], the precise pathogenesis of ALL is not clearly known. With the development of genetic pharmacology, people have gradually realized that individual differences in disease are mainly due to genetic polymorphisms, especially single nucleotide polymorphisms (SNPs) [5]. SNPs, a type of polymorphism involving variation of a single base pair, may directly affect the structure or expression of the protein. Therefore, the characterization and detection of the SNP distribution differences in different populations will help to identify human susceptibility genes related to some diseases.

Polymorphisms of the methylenetetrahydrofolate reductase (MTHFR) gene were shown to be associated with leukemogenesis [6]. MTHFR is a folate metabolic enzyme, catalyzing the reduction of 5,10-methylenetetrahydrofolate to 5-methyltetrahydrofolate [7,8]. Two common SNPs of the MTHFR gene, C677T and A1298C, have been identified. *MTHFR* C677T converts alanine into valine at codon 222, and A1298C converts glutamic acid into alanine at codon 429. Changes in these two loci can lead to decrease of enzyme activity and plasma folate level, as well as homocysteine level elevation [9]. Associations of the polymorphisms with a number of cancers, including prostate cancer, colorectal cancer, renal carcinoma and bladder cancer, have also been proposed [8,10]. Recently, the relativity between *MTHFR* polymorphisms and susceptibility to childhood ALL has come under intense scrutiny [2,6,11-16]. Because the *MTHFR* gene distribution varies among different populations [3,6], although relationships of *MTHFR* polymorphisms and leukemia in different populations have been reported [2,6,11-19], whether C677T and A1298C gene polymorphisms are involved in the susceptibility to childhood leukemia remains largely controversial.

In the present study, we used the polymerase chain reaction-restriction fragment length polymorphism (PCR-RFLP) technique to investigate whether *MTHFR* polymorphisms and their allele frequency may be correlated to susceptibility to childhood ALL among the Chinese population.

## Methods

### Patients

From October 2007 to April 2011, a total of 98 children (54 boys, 44 girls) with ALL were diagnosed by bone marrow cytology in Nanjing Children’s Hospital, Nanjing, China. Patient ages ranged from 0.9 to 11 years old (mean, 5.1 years old). Meanwhile, a total of 93 controls (1.2-13 years old; mean, 5.7 years old) hospitalized during the same period were recruited from clinical departments other than hematology or oncology of Nanjing Children’s Hospital. The controls had no blood disease or family history of ALL assessed by medical history and clinical examination. There was no significant difference between the two groups in gender and age distribution (*P* > 0.05). All individuals and/or their parents provided informed consent to participate in this study, and the protocol was approved by the medical research ethics committee of Nanjing Children’s Hospital.

### Genotyping of MTHFR variants

*MTHFR* C677T and A1298C polymorphisms were detected by PCR-RFLP assay as described by Atashrazm *et al.*[3] with slight modification. A total of 2 ml venous blood was extracted from each subject, and EDTA was used for anticoagulation. The conventional phenol-chloroform extraction method was used to extract the genomic DNA. *MTHFR* gene exon 4 (containing C677T polymorphism site) and exon 7 (including A1298C polymorphic site) were amplified by PCR. Primer sequences and the annealing temperature of PCR are presented in Table 1. For the details, the PCR thermal cycling condition included 35 cycles of 95°C for 5 min, 94°C for 30 s, 56°C or 55°C (for polymorphic sites at positions 677 and 1298, respectively) for 1 min, 72°C for 30 s and 72°C for 8 min. Primers were designed using Premier 5 software and synthesized by the Beijing Genomics Institute (Beijing, China). The primer’s specificity was confirmed by BLAST in Genbank.

**Table 1:** **Characteristics of PCR primers sequence, target fragment length and annealing temperature used for genotyping *MTHFR *** **polymorphisms**

**Polymorphisms sites**		**Primers sequence (5′ → 3′)**	**Fragment length**	**Annealing temperature**
*MTHFR* C677T	F	AGTCCCTGTGGTCTCTTCATC	387 bp	56°C
	R	GGAGATCTGGGAAGAACTCAG		
*MTHFR* A1298C	F	CTTTGGGGAGCTGAAGGACTACTAC	168 bp	55°C
	R	CACTTTGTGACCATTCCGGTTTG		

### Restriction endonuclease mapping analysis

*MTHFR* C677T: the PCR products were digested by HinfI (TaKaRa, Dalian, China) after overnight incubation at 37°C. The final products were subjected to electrophoresis on 2% agarose, and the genotypes were determined by examining the stained gel in 0.2% ethidium bromide. A fragment of 387 bp indicated a CC wild genotype; 3 fragments of 387, 235 and 152 bp were indicators of a CT heterozygote; a TT homozygous mutant produced two fragments of 235 bp and 152 bp, respectively.

*MTHFR* A1298C: PCR products were digested with MboII (TaKaRa, Dalian, China). The final products were subjected to electrophoresis on 2% agarose, and the genotypes were determined by examining the stained gel in 0.2% ethidium bromide. The AA wild genotype produced five fragments of 56, 31, 30, 28 and 18 bp, respectively; the AC heterozygote produced six of fragments 84, 56, 31, 30, 28 and 18 bp, respectively; the CC homozygous mutant produced four fragments of 84, 31, 30 and 18 bp, respectively. GENESCAN™672 software (Applied Biosystems, Foster City, CA, USA) was used for collecting information from every band, and GeneMapper software (Applied Biosystems, Foster City, CA, USA) was used to analyze the data.

### Statistical analysis

Data were input into Epidata3.0 to establish a database. Statistical analysis was performed by SPSS16.0 (SPSS Inc., Chicago, IL, USA). Hardy-Weinberg equilibrium was assessed by the χ^2^ goodness of fit test. Genotype and allele frequencies were expressed by percentile, and the haplotype and linkage disequilibrium were estimated by SHEsis software (Bio-X Life Science Research Center of Shanghai Jiao Tong University, Shanghai, China). The χ^2^ test was used to examine differences in *MTHFR* genotypes and allele frequencies between cases and controls. The odds ratio (OR) and 95% confidence interval (CI) were used for correlation analysis of ALL morbidity, and the *MTHFR* genotype. *P* < 0.05 was considered statistically significant.

## Results

### Hardy-Weinberg equilibrium test

Genotype distributions for both markers among control and ALL groups were found to be in HWE (Table 2).

**Table 2:** **Compositions of the *MTHFR *** **C677T genotype and *MTHFR *** **A1298C genotype in the Hardy-Weinberg equilibrium test**

	***MTHFR *** **C677T genotype**			***MTHFR *** **A1298C genotype**	
	**ALL group**	**Control group**		**ALL group**	**Control group**
CC type	28 (28.6%)	29 (31.2%)	AA type	54 (55.1%)	67 (72.0%)
CT type	44 (44.9%)	49 (52.7%)	AC type	42 (42.9%)	25 (26.9%)
TT type	26 (26.5%)	15 (16.1%)	CC type	2 (2.0%)	1 (1.1%)
χ^2^	1.013	0.569	χ^2^	3.652	0.643
*P-value*	0.314	0.451	*P-value*	0.06	0.423

### MTHFR C677T genotype and allele frequency distribution

Genotype and allele frequency for *MTHFR* C677T is shown in Table 3. Frequencies of 677CC, 677CT and 677TT were 28.6%, 44.9% and 26% in the ALL group and 31.2%, 52.7% and 16.1% in the control group, respectively. No significant differences in genotype distributions of *MTHFR* C677T were found between the ALL group and control group (χ^2^ = 3.109, *P* = 0.211). The 677 T allele frequency distribution between the two groups also showed no significant difference (49.0% vs. 42.5%, χ^2^ = 1.627, *P* = 0.202).

**Table 3:** **Frequencies of *MTHFR *** **C677T polymorphisms in childhood ALL patients and controls**

***MTHFR *** **C677T**	**Patient no. (%)**	**Control no. (%)**	**χ**^**2**^	***P***	**OR (95%CI)**
**Genotype**					
CC	28 (28.6)	29 (31.2)		-	1.00^#^
CT	44 (44.9)	49 (52.7)	0.046	0.829	0.930 (0.481 - 1.799)
TT	26 (26.5)	15 (16.1)	1.969	0.161	1.795 (0.790 - 4.079)
CT + TT	70 (71.4)	64 (68.8)	0.155	0.693	1.133 (0.609 - 2.106)
**Allele**					
C	100 (51.0)	107 (57.5)		-	1.00^#^
T	96 (49.0)	79 (42.5)	1.627	0.202	1.300 (0.868 - 1.947)

^#^As reference group; OR, crude odds ratio; —, not applicable; CI, confidence interval. CC, CT and TT overall distribution test, χ^2^_test_ = 3.109, *P* = 0.211.

### MTHFR A1298C genotype and allele frequency distribution

Genotype and allele frequency for *MTHFR A1298C* is shown in Table 4. Frequencies of 1298CC, 1298CT and 1298TT were 55.1%, 42.9% and 2.0% in the ALL group and 72.0%, 26.9% and 1.1% in the control group, respectively. The overall distributions of *MTHFR* A1298C genotype frequencies in the two groups were significantly different (χ^2^ = 5.917, *P* = 0.05). *MTHFR* 1298C allele frequency in the ALL group was significantly higher than that in the control group (23.5% vs. 14.5%, χ^2^ = 4.949, *P* = 0.026). Besides, from Table 4, we find that the OR of genotype CC is too large (0.219-28.104), which might result from the very low number of CC cases (two cases). Therefore, we combined the genotypes of AC and CC to calculate the distribution. The C allele (AC + CC genotype) frequency in the ALL group was significantly higher than that in the control group (44.96% vs. 28.0%, χ^2^ = 5.898, *P* = 0.015), and the C allele (AC + CC) carriers had a 1.1 times higher risk of ALL than the AA genotype carriers (OR = 2.100, 95% CI: 1.149 - 3.837).

**Table 4:** **Frequencies of *MTHFR *** **A1298C polymorphisms in childhood ALL patients and controls**

***MTHFR *** **A1298C**	**Patient no. (%)**	**Control no. (%)**	**χ**^2^*	***P***	**OR (95%CI)**
**Genotype**					
AA	54 (55.1)	67 (72.0)		-	1.00^#^
AC	42 (42.9)	25 (26.9)	5.628	0.018	2.084 (1.131 - 3.841)
CC	2 (2.0)	1 (1.1)	0.574	0.449	2.481 (0.219 - 28.104)
AC + CC	44 (44.9)	26 (28.0)	5.898	0.015	2.100 (1.149 - 3.837)
**Allele**					
A	150(76.5)	159 (85.5)		-	1.00^#^
C	46(23.5)	27 (14.5)	4.949	0.026	1.806 (1.068 - 3.053)

*Corrected chi-square test; ^#^as reference group; OR, crude odds ratio; —, not applicable; CI, confidence interval. AA, AC and CC overall distribution test, χ^2^_test_ = 5.917, *P* = 0.05.

## Discussion

In recent years, many articles have reported on the *MTHFR* polymorphism and susceptibility to ALL, but they are soundly inconclusive. In this study, by investigating the distribution of *MTHFR* C677T and A1298C genotypes and their allele frequencies in ALL children and non-ALL controls, we found that *MTHFR* A1298C was significantly related to the susceptibility to childhood ALL.

In 1999, Skibola *et al. *[20] first reported that the *MTHFR* C677T mutation could reduce the susceptibility to adult ALL, and their results suggested that the risk of ALL was reduced by 4.3 times for people carrying the TT genotype. Subsequently, Franco *et al. *[21] had similar findings that the C677T mutation can reduce the risk of childhood ALL. Later, multiple documents reported a protective effect of the TT genotype on the pathogenesis of ALL [3,6,11,12,]. However, the association is mostly not statistically significant [3,13], and some studies even suggested that the C677T mutation is an ALL risk factor [22]. A meta-analysis including 13 studies with a total of 4,894 individuals indicated that the C677T mutation was not associated with childhood ALL [23]. In this study, we also report a statistically non-significant difference of genotype frequencies among cases and controls, indicating that the *MTHFR* C677T gene polymorphism has no relation to the risk of childhood ALL. This result is in accordance with the results of Atashrazm *et al. *[3] and Oliveira *et al. *[13].

The *MTHFR* A1298C polymorphism was associated with an elevated risk of childhood ALL [6]. Alcasabas *et al*. [7] found that the ALL risk of *MTHFR* 1298C carriers (AC + CC type) was 1.57 times higher than that of the AA genotype carriers. *Kim et al. *[24,25] had similar findings. Other studies in Germany [17], the UK [2], north Indian [18] and Kurdish [19] showed no associations between the *MTHFR* A1298C polymorphism and risk of ALL. In our study, the risk of ALL in *MTHFR* 1298 AC genotype carriers was 2.084-fold that of the 1298AA genotype carriers (OR = 2.084, 95% CI: 1.131 - 3.841, *P* = 0.018), and the OR of the *MTHFR* A1298C genotype for AC + CC between patients and controls was 2.100 (95% CI, 1.149 - 3.837; *P* = 0.015). This indicates that A1298C is a risk factor for childhood ALL in the Chinese population, which is in accordance with the results of previous studies [6,7,24,26].

These data indicate that the *MTHFR* A1298C but not the C677T polymorphism is a potential biomarker for childhood ALL risk in the Chinese population, which is inconsistent with the results of Yan *et al. *[25] and Tong *et al. *[27]. We speculated that the inconsistent results in the relationship between *MTHFR* gene polymorphisms and childhood ALL in different studies might result from the following reasons: first, *MTHFR* gene distribution varied among different populations [6]; second, *MTHFR* polymorphisms may be affected by the level of intracellular folate, which is related to eating habits and nutritional intake; third, single SNPs provide insufficient information for comprehensive analysis of the *MTHFR* polymorphism; besides, other genes may regulate the susceptibility to childhood ALL, such as RFC1 80G > A and NNMT IVS -151C > T variants [12]. Eventually, linkage of haplotypes in the two loci would help to identify genetic markers associated with drug response differences.

The level of *MTHFR* 1298CC homozygous mutation is relatively low. In the present study, there were only two cases with CC genotypes among 98 patients. The previous studies found that the 1298CC homozygous mutation rate was only about 10% [3,12,18,20]. Thus, we speculated that the lower number of CC cases in this study was caused by the low total number of cases in this study (98 patients and 93 controls), which may induce a high occasionality. Therefore, we combined the genotypes of AC and CC to calculate the distribution of *MTHFR* A1298C to improve the reliability of our findings.

It is important to note that the small sample size is a major limitation in our study because only 98 cases and 93 controls were included. However, our study is still significant for providing valid data on *MTHFR* C677T and A1298C genotypes for further study of *MTHFR* polymorphisms and childhood ALL in the Chinese population. In addition, more case–control studies with larger sample sizes and more genotypes of different ethnic pediatric populations are necessary.

## Conclusion

In conclusion, we investigated the relationship between the polymorphism of *MTHFR* C677T and A1298C mutation and the susceptibility to childhood ALL by PCR-RFLP in 98 children with ALL and 93 non-ALL controls. The results indicated that the *MTHFR* A1298C polymorphism, not C677T, is associated with susceptibility to childhood ALL in the Chinese population. However, in the future, it will be necessary to expand the sample size and consider additional factors for exploring the relationship between the *MTHFR* polymorphism and the incidence of ALL to provide more accurate information for clinical research.

## Competing interests

The authors declare that they have no competing interests*.*

## Authors’ contributions

XL and QL participated in the design of this study. XL performed the statistical analysis. XL carried out the study, together with QL, collected important background information and drafted the manuscript. SZ collected blood samples and guided the experimental process. MC conceived this study, participated in the design and helped to draft the manuscript. All authors read and approved the final manuscript.
